# Micro Computed Tomography and Immunohistochemistry Analysis of Dental Implant Osseointegration in Animal Experimental Model: A Scoping Review

**DOI:** 10.1055/s-0042-1757468

**Published:** 2023-03-28

**Authors:** Annisa Putri, Farina Pramanik, Azhari Azhari

**Affiliations:** 1 Dentomaxillofacial Radiology Residency Program, Faculty of Dentistry, Universitas Padjadjaran, Bandung, Indonesia; 2 Department of Dentomaxillofacial Radiology, Faculty of Dentistry, Universitas Padjadjaran, Bandung, Indonesia

**Keywords:** dental implant, osseointegration, animal model, micro-CT, immunohistochemistry

## Abstract

Osseointegration is a complex process that involves the interaction of dental implants, bone, and the immune system. Preclinical testing was carried out to develop a better understanding of the mechanism. Micro-computed tomography (micro-CT) imaging techniques and immunohistochemistry are excellent tools for this objective as both enable quantitative assessment of bone microarchitecture and intercellular interaction. An extensive literature search was conducted using the databases PubMed, Science Direct, Wiley Online, Proquest and Ebscohost from January 2011 to January 2021. Among the publications retrieved, the rat model was the most frequently used experimental protocol, with the tibia being the most frequently implanted site. The region of interest demonstrates a high degree of homogeneity as measured by trabecula but varies in size and shape. The most frequently mentioned micro-CT bone parameter and immunohistochemistry bone markers were bone volume per total volume (BV/TV) and runt-related transcription factors (RUNX). Animal models, micro-CT analysis methods, and immunohistochemistry biomarkers yielded a variety of results in the studies. Understanding bone architecture and the remodeling process will aid in the selection of a viable model for a specific research topic.

## Introduction

Dental implant osseointegration is classically described as direct contact between a living bone and an implant material at a light microscopic level. More recently, however, the definition combines several viewpoints of a process involving microstructural and immunomodulation of bone tissue regeneration. 1 2 Osseointegration aims to maximize the implant-to-bone contact while lowering failures. 3 4 To further understand the mechanism, *in vivo* studies have been used to conduct preclinical testing, but there are debatable subjects such as which animal models (large animal or rodent) could strongly correlate with clinical outcomes, particularly relevant to human maxillofacial intramembranous ossification and accommodating the biomechanical properties (size, design, topography, and drilling site) of the implant. 5

Micro-CT allows comprehensive examination of three-dimensional microstructures of a bone in tiny samples. This methodology has been verified and is now used to measure bone microarchitecture as the new gold standard method. 6 7 Previous studies mainly investigated only the whole bone mass; so, there is much inadequate information regarding which specific region of interest (trabecula or cortical or marrow) of the bone surrounding the implant reflects osseointegration. Furthermore, micro-CT can also be used to measure various osseointegration-related bone histomorphometrics. 8 9 10

While evaluating osseointegration, more attention needs to be paid not only to the microstructural changes but also to the bone immune microenvironment. 11 Following implant placement, several intercellular reactions occur for bone repair. Immunohistochemistry was used to evaluate the reactions, which play an important role in the osteogenic activity from osteoblast differentiation to organic matrix synthesis, mineralization, and bone remodeling. Immunohistochemistry was conducted to analyze by observing the intense activity of specific protein or biomarker expression. 12 13

Therefore, the aim of this study was to identify through a screening of scientific literature how micro-CT and immunohistochemistry analysis are being applied in animal models for the investigation of dental implant osseointegration. In addition to this general objective, specific questions were formulated: (i) In animal model experimental, which species are suitable for dental implantation, and how long the osseointegration process would be evaluated? (ii) In micro-CT analysis investigating the bone around the implant, what are the appropriate regions of interest, and which bone parameters can be quantified? (iii) In immunohistochemistry analysis investigating the osteoimmunity process during osseointegration, which biomarkers are representable to be measured?

## Methods

Original articles related to the topic were searched in six databases (PubMed, Science Direct, Wiley Online, ProQuest, and EBSCOhost) published from January 2011 up to January 2021 using the following keywords and MeSH terms stated on Boolean operators: ((X-ray microtomography” OR microtomography OR “micro-CT”) AND (“dental implant” OR implant) AND (“gene expression” OR “RT PCR” OR immunohistochemistry)) NOT (“stem cell” OR “culture cell” OR “in vitro” OR orthopedic). The following criteria were used to determine the eligibility for this review: *in vivo* experimental studies with therapeutic intervention (drug-induced, systemic disease, mechanical testing), a study performed at least one micro-CT and one immunohistochemistry analysis, and full-text articles. There was no limitation regarding heterogeneity and sample size. Meanwhile, articles published in non-English languages were excluded. Micro-CT analysis using linear measurement and immunohistochemistry analysis using histomorphometry count were also excluded. The searched publications were only considered in the English language.

**Table 1:** 

No.	Author and year	Animal model (sex, age, species)	Observation time point	Implant Site	Region of Interest (size and shape)	Micro-CT bone parameter	Immunohistochemistry marker/protein
1.	(Vandamme et al, 2011) 14	Female, 14-week old, Janvier Wistar rat	9 days	Tibia	3.38 mm in length	BV/TV, Tb.Th, Tb.Sp, Tb.N	IL-11
2.	(Diao et al, 2017) 27	Female, 20–22 week old, New Zealand rabbit	0 days, 7 days, 14 days, 28 days	Femur	0.5 mm around the implant and 5.5 mm in length	Tb.N, Tb.Th, Tb.Sp	Wnt/β-catenin, RANKL
3.	(Tan et al, 2017) 15	6 months, mini pig	3 months, 6 months	Mandible	0.2 mm around the implant	BV/TV, Tb.N, Tb.Th, Tb.Sp	RUNX2
4.	(Yi et al, 2017) 31	Female, 8-month-old, Sprague–Dawley rats	8 weeks	Femur	1 mm around the implant	Tb.N, Tb.Sp, Tb.Th	RUNX2, OPN
5.	(Biguetti et al, 2018) 25	Male, 10-week-old, wild-type mice (C57Bl/6)	3 days, 7 days, 14 days, and 21 days	Maxilla	500 µm axis in length and 100 µm from the implant in width	BV/TV	BMP2
6.	(Cirano et al, 2018) 17	Male, 10-week-old, Wistar rat	30 days	Calvaria	Between the 1st and last threads (214 slices)	BV, Tb.Th	BMP2, OPN, RANKL, RUNX2, Wnt/β-catenin
7.	(Faverani et al, 2018) 18	Female, 10-week-old, Rattus norvegicus albinus Wistar rat	42 days	Tibia	Rectangular area, 0.5 mm in length and 0.8 mm in width between the 3 ^rd^ and 5 ^th^ (100 slices)	BV/TV, Tb.Th, Tb.Sp and Tb.N	RANKL
8.	(Freitas de Paula et al, 2018) 19	*Rattus norvegicus* Albinus variation	15 days, 30 days, 60 days	Tibia	Rectangular area, 0.5 mm in width	BV	OCN
9.	(Liu et al, 2018) 20	Female adult Sprague–Dawley rats	3 days, 7 days, 21 days	Mandible	Circular area, 0.5 mm in width	BV/TV, Conn.D, Tb.Th, Tb.N	Wnt/β-catenin, RUNX2
10.	(Palin et al, 2018) 21	Male, 3-month-old, *Rattus norvegicus* albinus Wistar rat	60 days	Tibia	0.5 mm in width	Po(tot), Conn.Dn	RUNX2
11.	(Pinotti et al, 2018) 41	12-week-old, *Rattus norvegicus* variation Hotzman rat	45 days	Tibia	Circular area, 0.5 mm in width	BIC; BBT	OCN, BMP2
12.	(Yogui et al, 2018) 22	4-month old, *Rattus norvegicus* variation Albinus rat	14 days, 42 days, 60 days	Tibia	Rectangular area, 0.5 mm in length and 0.8 mm in width between the 3 ^rd^ and 5 ^th^ threads	BV, Po.N, Po.V, Po(tot)	RUNX2, OPN, Wnt/β-catenin
13.	(Li et al, 2019) 26	Male, 4-week old, C57BL/6J wild-type mice	6 weeks	Maxilla	Cylinder area, 1.0 mm in width and 1.0 mm in length	BV/TV	IL6
14.	(Ribeiro et al, 2019) 23	Male, 10-week-old, Wistar rat	30 days	Tibia	Between the 1st and last threads (214 slices)	BV, Po(tot), Tb. Sp, Tb.Th	BMP2, OPN, RUNX2, Wnt/β-catenin, RANKL
15.	(ZHU et al, 2019) 28	Male, adult, New Zealand *Oryctolagus cuniculus* Rabbits	3 weeks, 5 weeks, 12 weeks	Mandible	1 mm in width around the 3 ^rd^ and last thread	BV/TV, Tb.Th, Tb.N, Tb. Sp	BMP2, OPN, RANKL
16.	(Corrêa et al, 2020) 24	Male, 10-week-old, Wistar rats	30 days	Tibia	Between the 1st and last threads (214 slices)	BV, Tb.Th	BMP2, OPN, RANKL, RUNX2
17.	(de Oliveira et al, 2020) 16	Wistar adult	7 days and 30 days	Tibia	1 mm in width	BV/TV, Po(tot)	RUNX2, OPN, RANKL
18.	(dos Santos Trento et al, 2020) 13	Male, 5 months old, *Oryctolagus cuniculus*	15 days, 30 days, and 60 days	Tibia	Circular area, 0.5 mm in width	Tb.N, Tb.Sp, Tb.Th, Conn.Dn	RUNX2, OPN, RANKL
19.	(Ye, Huang and Gong, 2021) 29	Male, 15 months old, Beagle dogs	2 weeks, 4 weeks and 8 days	Mandible	Between the 1st and last threads	Tb.Th, Tb.N, Tb.Sp	RUNX2, OPN, Wnt/β-catenin

Abbreviations: BBT, bone between thread; BIC, bone in contact; BMP2; bone morphogenetic protein; BV, bone volume; Conn.D, connectivity density; IL-1, interleukin 1; IL-6, interleukin 6; OCN, osteocalcin; OPN, osteopontin; Po(tot), total of porosity; Po.N, number of pores; Po.V, volume of pores; RANKL; Receptor activator of nuclear factor kappa-Β ligand; RUNX2, Family Transcription Factor 2; Tb.Sp, trabecula separation; Tb.N, trabecula number; Tb.Th, trabecula thickness; TV, total volume.

## Animal Experimental Model and Dental Implantation Site

Varies of animal models were found using rats, 14 15 16 17 18 19 20 21 22 23 24 mice, 25 26 rabbits, 13 27 28 beagle dogs, 29 and minipigs. 15 The results of this scoping review are shown in Table 1 . The literature research identified rat models are the most reported protocol to be applied, the species have several advantages including 99% similarity to the human genome, availability of several efficient genetic or molecular tools, the animal's small size facilitates the use of reduced quantities of drugs and reduced experimental period. The rat model has been adopted for a long time although mostly for extra-oral procedures due to technical and surgical challenges, with the most frequently reported cause being the difficulty of access due to the mouth size and range of opening of mice. At least one implant per tibia can be evaluated using a nearly human-size implant (2.0 mm in diameter and 4.0 to 5.0 mm in length). Bi-cortical anchoring is also possible with this model. A diameter of 1.5 mm and a length of 2.5 mm are highly acceptable for multi-implant techniques. 30

The animals' ages ranged from 4 weeks to 15 months and male animals were preferable. Tibia 13 14 16 18 19 21 22 23 24 is the primary implant site, followed by the maxila 25 26 and the mandible, 15 20 28 29 femur, 27 31 and calvaria. 17 Furthermore, long skeletal bones such as the tibia and femur were the most prevalent site to insert the implant compared with the maxilla or mandibula. In this context, osseointegration in endochondral bones is achieved through the program of endochondral ossification, which differs from osseointegration in the maxillofacial. In addition, there is a large proportion of marrow cavity in the implantation sites of long bones, which exhibit the slowest reaction to implant placement compared with the periosteum region. Therefore, while these studies are useful to better understand the osseointegration process in orthopedics applications, they cannot be fully translated for the context. 25

## Time Point to Follow-up Dental Implant Osseointegration

The time between implant placement and osseointegration monitoring ranged from 0 to 6 months. The most common analysis period was 30 days. 13 16 17 19 23 24 Other studies 13 19 20 22 25 27 28 conducted multiple observation varies up to four difference periods. A Uniform time point is hardly be achieved and a parallel comparison of the biological process of osseointegration is difficult to determine. In a long skeletal protocol, 2 to 6 weeks are needed before assessing osseointegration. In the case of implant placement at a healed extraction site, 1.5 months of healing is generally allowed after extraction and another month for implant osseointegration. In the maxilla, protocols are shortened, with implantation performed immediately after extraction. 30 The average healing period following implant placement was 13.4 to 28 weeks for submerged implants and 13.2 to 40 weeks for nonsubmerged implants. 32 Other factor to consider is the high cost inherent to animal studies, which is undoubtedly an impeding factor to prospective researchers. The time required for the natural progression of osseointegration in animal models vastly increases the animal feeding and housing costs, as well as surgical costs and maintenance personnel fees. 33

## Micro-CT Analysis

Radiographic examinations from the moment of implant placement were necessary to examine the first bone remodeling, which can be caused by surgical stress or soft and hard tissue homeostasis. 34 Micro-CT analyzes basic parameters (bone volume [BV] and total volume of interest [TV]) 14 15 16 17 18 19 20 24 25 26 28 as well as trabeculae microstructural features such as (trabecular thickness [Tb.Th], trabecular separation [Tb.Sp], trabecular number [Tb.N]), 13 14 15 18 27 28 29 31 connectivity density (Conn.Dn), 13 20 21 and total porosity percentage (Po[tot]), 21 number of pores (Po.N), and volume of pore (Po.V). 22 BV/TV can offer an objective indicator for bone mineral density in the implant area, which is crucial for assessing initial implant stability. 35 Only specific trabecular bone parameters such as BV/TV and Tb.Th are affected by scanning parameters when reconstructing images using larger voxel sizes. This is because the trabecular bone parameters are significantly affected by the scanning voxel size rather than the reconstruction voxel size. 36

The region of interest considered for analysis showed great homogeneity focused on evaluating trabecula. According to Lekholm and Zarb, implant placement in type 1 (homogenous cortical bone), type 2 (thick layer of cortical bone surrounding a central part of a dense trabecular bone), and type 3 (thin layer of cortical bone surrounding dense trabecular bone of favorable strength) bone results in good clinical outcomes. Trabecular bone has a greater turnover than cortical bone because it contains bone marrow, which is the source of osteoblasts and osteoclasts. The structure of trabecular bone appears to play a little role in primary implant fixation, but it is critical for peri-implant bone repair. 37 38 39

The included studies showed different sizes and shapes to determine the region of interest, rectangular area, 18 19 22 circular/cylinder area, 13 19 20 26 and the rest studies had customized contours. The majority of studies measured the osseointegration area vertically starting from the most coronal to the most apical dental implant reaching the entire length or diameter, while three studies 18 22 28 analyzed only from the third thread to the fifth thread. The measurement also occupies 0.2 mm 15 or 0.5 mm 19 20 21 27 or 1 mm 14 26 28 31 area horizontally from the margin or the outer surface of the implant.

The differences between the studies were concerned with selecting the best area to represent great osseointegration. Most studies applied the distance of 0.5 mm to 1 mm of the surrounding implant, due to bone remodeling is the greatest in the bone adjacent to the interface (within 1 mm of the implant) and decreases with the increasing distance from the implant, according to a histomorphometry comparison in four species including humans. 40 However, other studies chose the middle and lower two-thirds of the implant as the region of interest (ROI) because those areas were more closely contacted by the surrounding alveolar bone after immediate implant placement. 28

## Immunohistochemistry and Immunofluorescence Analysis

We analyzed through an exploratory real-time polymerase chain reaction array and immunostaining considering the molecules involved in the inflammatory response and bone healing (growth factors; immunological/inflammatory markers; extracellular matrix, MSC and bone markers) to select targets with a significant expression. The immunohistochemical evaluation was performed using ordinal qualitative analysis, in which immunostaining for several proteins involved in the bone formation process was scored. Early bone formation markers RUNX2, late bone formation markers, and remodeling markers RANKL were found to be upregulated in the osseointegration process. 25 *RUNX2* is also an important gene for osteoblast differentiation and function. 13 These specific proteins represent the earliest stages of the bone healing process at 60 days. 21 Mutations in genes associated with lipoprotein receptor-related proteins (LRPs) have been shown to reduce osteoblast numbers and favor the onset of osteoporosis, highlighting the role of canonical Wnt/catenin signaling in bone tissue pathogenesis. Wnt/catenin and RUNX-2 osteoblastogenesis biomarkers are more expressive at 14 days, while osteopontin and osteocalcin are more expressive at 42 days. Immunofluorescence and RT-qPCR were used to investigate sclerostin, -catenin, and RANKL during bone remodeling. No substantial change in the cortical bone around the implant was identified, however debonding at the interface and decreased osseointegration were. Sclerostin, -catenin, and RANKL expression correlates with bone damage and remodeling. Based on this, Immunofluorescence analysis can determine the osteoimmunity process during osseointegration by staining proteins that play a role in bone damage and remodeling. Moreover, further analysis can evaluate possible osseointegration pathways. These results suggest that sclerostin regulates the Wnt/-catenin and RANKL/RANK pathways to affect bone growth and resorption. 20 27 At 60 days, there was no specific cellular expression due to bone maturation. 22

## Conclusion

This study shows heterogeneous results from animal models, methods of micro-CT, and immunohistochemistry analysis. While there is no standard procedure that meets all the characteristics of an ideal preclinical model, an understanding of bone architecture and the bone remodeling process will aid in the selection of a model that is appropriate for a specific research issue.
